# Leptin in Human Milk—One of the Key Regulators of Nutritional Programming

**DOI:** 10.3390/molecules27113581

**Published:** 2022-06-02

**Authors:** Elena Sinkiewicz-Darol, Iwona Adamczyk, Katarzyna Łubiech, Gabriela Pilarska, Magdalena Twarużek

**Affiliations:** 1 Department of Physiology and Toxicology, Faculty of Biological Sciences, Kazimierz Wielki University, Chodkiewicza 30 St., 85-064 Bydgoszcz, Poland; iwonaadamczyk25@gmail.com (I.A.); gabrielapilarska@ukw.edu.pl (G.P.); twarmag@ukw.edu.pl (M.T.); 2 Human Milk Bank, Ludwik Rydygier Provincial Polyclinical Hospital, St. Josef 53-59, 87-100 Torun, Poland

**Keywords:** leptin, breast milk, obesity

## Abstract

Breast milk is the optimal food for infants and toddlers, providing basic nutrients. It is also a source of many biologically active substances. Among them are hormones responsible for metabolic balance. One of the hormones taken in with breast milk by a breastfed baby is leptin. This hormone is involved in the regulation of appetite, informing the brain about the body’s energy resources. Having the correct mechanisms related to the action of leptin is a factor reducing the risk of obesity. The natural presence of leptin in the composition of breast milk suggests that it has a specific role in shaping the health of a breastfed child. Obesity as a disease of civilization affects more and more people, including children. The development of this disease is multifaceted and determined by many factors, including genetic and environmental factors such as eating habits and low physical activity. Behind obesity, there are complex mechanisms in which many elements of the human body are involved. Understanding the effects of breastfeeding as a natural source of leptin can help prevent childhood obesity and development of this disease in future life.

## 1. Introduction

Human milk (HM) is characterized by a variety of components. Fluctuations in the content of macronutrients are observed both in the daily cycle and throughout the entire lactation period [1]. Changes in the content of individual nutrients result from the changing needs of a growing child. The composition of HM responds to these needs and adapts to meet the nutritional requirements of the infant [2]. Apart from changes in the composition of macronutrients, the content of biologically active factors in breast milk is also not constant [3]. It depends on a number of both maternal and environmental factors as well as the needs of the breastfed child.

Obesity is a civilization disease that affects a growing group of society. There is an increasing number of children suffering from overweight and obesity [4]. The most frequently recognized factors that contribute to the emergence of obesity include incorrect eating habits and too little physical activity, but genetic predisposition is also mentioned [5]. Research has confirmed the link between overweight in childhood and overweight in adulthood. It is also associated with an increased incidence of cardiovascular diseases and diabetes in later life [6].

Many researchers have focused on the importance of early-life nutrition in terms of predisposition to obesity and overweight. Those early stages also include breastfeeding. Studies have shown a reduced risk of obesity later in life in breastfed infants [7]. Despite many optimistic scientific reports on the effects of breastfeeding on obesity prevention, this issue is very complex and often questioned [8,9]. This is due to the ambiguity of the results and the multifactorial impact on the child’s development. It is difficult to investigate the influence of individual factors on the health status of a child, mainly due to individual differences in the composition of human milk. Although many studies have shown the protective effect of breastfeeding, a question must be asked about the strength of this effect and its contribution to the protection against obesity. 

Leptin, a hormone that is present in human milk and regulates food intake and energy metabolism, is recognized as playing a role in the postnatal programming of a healthy phenotype in adulthood [10].

## 2. Materials and Methods

Interventional studies involving animals or humans and other studies that require ethical approval must list the authority that provided approval and the corresponding ethical approval code. The methodology to perform this review included the following processes: definition of the aim of the review, a literature search, data collection, evaluation, comparison and analysis. The literature review included electronic searches of Ebsco, Web of Science, PubMed and ScienceDirect. A bibliographic search strategy was conducted to identify studies reporting on leptin in human milk and its influence on the risk of obesity. The electronic search used the following terms: breast milk, leptin, leptin gene, obesity, variability in breast milk composition, breast milk ingredients, infant nutrition, physiology, sequence variant, single nucleotide polymorphism. The reference lists of the previous reviews and relevant studies were examined. The inclusion criteria were papers written in English published between January 1997 and March 2022. The quality of controlled studies was critically appraised.

## 3. Leptin—Characteristics of the Hormone

Composed of 167 amino acids, leptin is a polypeptide hormone, the product of the ob (LEP) gene, with a molecular weight of about 16kDa. The LEP gene was first identified and described in 1994 by Zhang et al. In humans, the LEP gene is located on the long arm of chromosome 7 (7q31.3) and consists of three exons and two introns. The gene expression shows diurnal fluctuations. Leptin receptors belong to class I cytokines. The intracellular leptin-binding domain shows strong homology to gp130 subunits that signal to IL-6, G-CSF and LIF receptors. These receptors, like the leptin receptor, bind to Janus kinase (JAK) and phosphorylate tyrosine on the cytoplasmic portion of the receptor, to which STAT 3 (signal transducer and activator of transcription 3) is recruited (Heinrich et al., 2002). The concentration of leptin in serum is directly proportional to the amount of white adipose tissue (WAT) or food intake [11,12]. 

Leptin is released as a proteohormone by white adipose tissue and, to a lesser extent, by the gastric mucosa, ovaries, testicles, pituitary gland, vascular endothelia, skeletal muscles, brown adipose tissue and the thoracic gland [13,14].

The activity of leptin is pleiotropic, as it regulates the amount of body fat, affects body weight and is linked with a number of other processes, such as the regulation of energy homeostasis, blood pressure, hematopoiesis, reproduction-related processes (regulation of GnRH, FSH and LH), angiogenesis and the functions of the immune system [15,16,17]. Leptin exerts all of its effects through its specific receptors (OB-R). Initially, it was thought that the biological effect of leptin was to stimulate receptors located in the hypothalamus [18]. However, it is now known that these receptors are also found in other tissues, including adipose tissue, stomach, spleen, liver, lungs, heart, thymus, mammary gland, placenta, endometrium, ovaries and testes [19]. In the hypothalamus, the biological activity of leptin consists in the principle of the negative feedback loop between the concentration of this hormone in the blood serum and the expression of its receptor [20]. This is mainly manifested by a decrease in appetite, an increase in energy expenditure and the activation of the sympathetic nervous system owing to the inhibition of the neuropeptide Y secretion responsible for increasing appetite [17,21]. The regulation of weight gain is conditioned by both the correct structure of leptin itself and the functionality of its receptors. 

Studies on animal models and in obese individuals show an increase in leptin concentration in circulation [12,22]. This may be related to the insensitivity of leptin receptors, reduced receptor expression or hampered signal transduction in the hypothalamus. Moreover, it is also connected with impaired leptin transport in blood in adulthood and in obese individuals. Resistance to leptin, impairment of the regulatory outflow of leptin and increased food intake contribute to the impairment of energy homeostasis, which exacerbates the problem of obesity and has serious consequences for the whole body. The reason for this situation is complex and has not been fully understood yet [12,17]. Leptin is secreted in a pulsatile diurnal rhythm, with a peak at night. Its concentration is approximately 30–100% higher at night than in the morning. Leptin concentration is reflected not only in the amount of accumulated carcass tissue but also in a person’s energy balance, but the energy balance of the body does not solely depend on leptin. Apart from leptin, ghrelin, adiponectin, epidermal growth factor (EGF) and insulin-like growth factor 1 (IGF-1) also contribute to the formation of adipose tissue and lean body mass. Leptin, adiponectin and ghrelin affect appetite regulation and energy balance. EGF and IGF-1 are trophic factors that influence growth, differentiation and proliferation of cells. The presence of these hormones regulates the state of nutrition and energy homeostasis [23,24].

## 4. Molecular Effect of Leptin

The pleiotropic distribution of LEP-R facilitates the action of leptin; its binding to the receptor initiates the signal transduction pathway and consequently regulates a number of cellular functions in the body. LEP-R belongs to type I cytokine receptors and transduces the signal via Janus tyrosine kinases (JAKs) [25].

Signal transduction pathways are responsible for mediating the metabolic effects of leptin on the cardiovascular system. As an example, the JAK2/STAT3 pathway is responsible for regulating changes in gene expression when the PI3K pathway transduces the signal more rapidly through phosphorylation of cytoplasmic proteins. The PI3K pathway is important during acute effects on leptin, being responsible for regulating food intake and hypertension. However, the JAK/STAT3, MAPK and also PI3K pathways are collectively responsible for the regulation of energy balance [26].

Leptin acts similarly to other reactive acute phase factors, causing increased secretion of many inflammatory cytokines such as IL-6, IL-12 and TNF-α. On the other hand, exposure to inflammatory stimuli such as TNF-α and IL-2 increases leptin expression in adipose tissue. This leads to a feedback loop that promotes inflammation. This loop shows how leptin promotes low-grade inflammation (pro-inflammatory mediators cause increased expression of leptin and other acute phase reactive factors that promote chronic inflammation) [27]. The effects of leptin are manifold: it stimulates the expression of IL-1Rα, cluster of differentiation (CD) 25, CD39, CD69 and CD71 and the production of the pro-inflammatory cytokines TNF-α and IL-6 in macrophages [28]. The amount of macrophages present in adipose tissue correlates directly with obesity, as obese individuals have more macrophages. Cytokines produced by adipocytes, namely CC-chemical ligand 2 (CCL2), affect the infiltration process of macrophages. Macrophages as well as adipocytes in adipose tissue are the source of TNF-α and IL-6 in obese individuals [29]. Thus, adipose tissue cells are also involved in a feedback loop that perpetuates the production of pro-inflammatory cytokines. These feedback loops explain why obesity is associated with chronic pro-inflammatory signaling pathways and with abnormal cytokine production and increased acute phase reactive factors and show why obesity increases the risk of developing inflammatory diseases and why it leads to immune dysfunction [30].

## 5. Leptin in Human Milk

To date, there are few studies on the leptin content in human breast milk and its potential effects on weight control in developing infants. The collected data related to the concentration of leptin in milk are presented in Table 1.

All of the studies presented below involved testing leptin levels in skimmed milk. However, the methodology used was often divergent, and the results show a wide range of leptin concentration levels in each individual study. Hence, direct comparison of the studies seems to be a difficult task. 

Some of the studies highlight the effect of maternal BMI during pregnancy on the leptin content in milk. Logan et al. indicated that the leptin concentration in milk in the 6th week and 6th month and after one year of breastfeeding (n = 668, n = 445 and n = 69, respectively) is non-linearly related to the maternal BMI [10]. Chan et al. (2018) emphasized the strong correlation between maternal BMI before delivery and leptin concentration in milk as well as lower leptin concentration in milk in multiparous and older women [32]. In addition, a high concentration of leptin in milk was related to lower weight-for-length and BMI of a child. A similar relationship was observed by Fields et al. [3] and Dadres et al. [33], who showed that the leptin concentration in milk was statistically significantly correlated with maternal BMI before pregnancy or maternal BMI after delivery, but it was not correlated with children’s body weight [40]. Bronský et al. (2006) [31] demonstrated that leptin was significantly lower in mothers who delivered preterm vs. term infants. Interestingly, this study also showed that leptin concentrations were higher in mothers who delivered boys as opposed to girls, but the differences were not considerable. The studies by Brunner et al. [35] and Schister et al. [38] showed similar concentrations of leptin in milk. However, neither of them demonstrated that changes in leptin concentration affected the development and body build of the examined children up to 2 years or 6 months of age, respectively. What is more, no correlation between leptin concentration in milk and maternal BMI before pregnancy was observed [10,41]. The study by Miralles et al. [6] involving a group of 28 non-obese women did not show any significant correlations between leptin concentration in milk and a newborn’s body weight [1]. The study by Khodabakhshi et al. [23] did not demonstrate that leptin concentration in milk was statistically correlated with overweight or obesity in a child. In the study by Galante et al. [42], the authors examined the correlation of leptin concentration in milk 2.6 ± 0.4 months after delivery and the development of children between 13 months and 5 years of age. According to the researchers, leptin concentration in milk was not related to the height of a child at a later time. In the studies comparing leptin concentrations in foremilk and hindmilk by Karatas et al. [34] and Nikanorova et al. [14], the authors showed a difference where leptin concentrations were higher in hindmilk in the group of children breastfed between 1st and 3rd months of age and between the 4th and 6th months of age, but the differences were not statistically significant. Similar results were obtained by Schueler et al. [39] and Reid et al. [13], who compared leptin concentrations in foremilk and hindmilk. The differences were not statistically significant, but leptin concentration showed a correlation with maternal BMI, body weight and body fat content in a mother. Savino et al. (2002) [36] demonstrated statistically significant higher leptin concentrations in the serum of exclusively breastfed children in comparison with children fed only with infant formula, which can be related to the delivery of leptin with milk in breastfed children and the potentially protective effect of human milk. In another study, the same author examined the leptin concentration in milk, which showed no correlation with anthropometric parameters of infants.

The above-mentioned data do not allow to draw clear conclusions in relation to the content of leptin in human milk and its effect on the development of a child. Further studies are necessary to assess the effects of leptin in human milk on the development of a breastfed child.

## 6. The Effect of Leptin on Nutritional Programming in Infants and Toddlers

Research has shown a positive correlation between the content of leptin in breast milk and the maternal body mass index (BMI). There is a positive relationship between maternal weight, BMI and skin fold thickness [2,43,44]. The research carried out by De Luca et al. [45] showed that the leptin concentration in the milk of obese mothers was twice as high as that in the case of mothers with normal body weight. The concentration of leptin in breast milk is correlated with the level of leptin in the mother’s plasma. 

However, a negative correlation can be observed when analyzing the relationship between the content of leptin in human milk and the growth of breastfed infants [6]. Exclusively breastfed infants born to obese mothers showed lower infant weight gain in the first month of life [45]. The studies conducted by Uysal et al. [40] showed no relationship between the concentration of leptin in the breast milk of mothers breastfeeding obese and non-obese infants. These results contradict the theory that breast milk leptin has a significant effect on obesity in infancy.

Gastric emptying takes place at a controlled rate and influences nutritional behavior by regulating the amount of food consumed. Studies conducted by Gridneva et al. [46] did not show a relationship between the concentration or dose of leptin consumed by breastfed toddlers and the rate of gastric emptying.

Taking into account the influence of leptin on the body in the initial period of nutrition, it is important to develop the best nutritional solutions. Considering the resulting consequences for children’s health, the lack of leptin in formula is puzzling. What is more, infants fed with human milk from human milk banks may be at risk of deficiency in the consumption of exogenous leptin. The pasteurization process, which is commonly used in human milk banks, contributes to a decrease in the level of leptin in women’s milk [47]. However, a comparative study of serum leptin levels in breastfed and formula-fed infants by Lönnerdal et al. [41] did not show any significant differences in leptin levels in blood. In contrast, pasteurization of formula milk has no effect on the decrease in leptin. This may indicate that exogenous leptin does not contribute greatly to the maintenance of infant serum leptin levels. The opposite results were obtained by Savino et al. (2002) [36]. In their studies, the serum levels of leptin in breastfed newborns were higher than those in formula-fed toddlers.

Nutrition in early life—i.e., both the nutrition of a fetus during pregnancy and the nutrition of a newborn—has a significant impact on the energy processes that occur later in life. Even the availability of nutrients can affect the functioning of the body in the future. The conducted studies indicate the influence of low birth weight on the incidence of obesity in adulthood. This is due to the altered body composition proportions of the organism experiencing nutritional deficiencies [48].

Despite the controversy related to the influence of leptin contained in human milk on a child’s body, a number of studies showed that breastfed infants are less likely to become obese later in life compared to formula-fed babies. The length of breastfeeding is also important. Long breastfeeding is beneficial in preventing obesity. Smaller weight gain in breastfed babies may be due to the fact that breastfed babies eat smaller portions but more often. Additionally, the total average amount of energy absorbed by breastfed children is lower than the average number of calories consumed by formula-fed children [49].

Research indicates that the early stage of a child’s life is crucial in the process of preventing obesity. Nutritional intervention based on the application of correct nutritional practices is involved in nutritional programming [50]. This applies to the influence of bioactive factors but also to the formation of the correct profile of the gut microbiota [51,52,53]. We can observe an increasing number of obese children, especially in high-income countries. At the same time, research shows that in these parts of the world, the period of breastfeeding is shorter and the breastfeeding practices are generally not in line with WHO recommendations for exclusive breastfeeding for six months and continued breastfeeding for up to two years [53,54,55]. One of the studies conducted showed that each subsequent month of breastfeeding resulted in an additional 4% decrease in the risk of obesity later in life [56]. Therefore, it is necessary to take into account the potential relationship between the problem of short-term breastfeeding and the prevalence of obesity among new generations.

On the other hand, although the active compounds in breast milk have a protective effect on obesity, overfeeding a child even with human milk may result in an increased risk of obesity in later childhood [6]. At the same time, numerous studies have failed to show the preventive effect of breastfeeding on obesity. A meta-analysis on the effects of infant breastfeeding on BMI in later life showed no significant differences between breastfed and formula-fed individuals. Although breastfeeding may have a protective effect on obesity, there is also a significant share of other factors determining the occurrence of obesity. The protective effect of breastfeeding may thus turn out to be insufficient when confronted with the whole range of predisposing factors of obesity [57].

The optimal dose of leptin ingested with milk to best benefit infants has not been established. The probable mechanism of action of milk leptin has mainly been indicated in studies with the use of animal models. To date, several forms of leptin have been demonstrated. Gastric leptin is released into both the gastric lumen and the bloodstream. Hence, gastric leptin may contribute to local effects in the stomach and to the accumulation of circulating leptin, thus exerting a systemic effect [58,59]. Studies have shown that the increase in circulating leptin from the gastric epithelium is probably not large enough to produce a systemic effect in comparison to leptin from adipose depots. It is suggested that leptin works mainly locally, e.g., by signaling a satiety signal. Thus, adipocyte-derived leptin and stomach-derived leptin are identical molecules, but they have different roles in food intake control [59]. Studies on animal models have shown that the production of gastric leptin in the neonatal period does not appear to be fully efficient during most of the lactation period, when the stomach is still immature. Leptin mRNA levels in the stomach of neonatal rats were low during the first days of life and increased from day 15, which was associated with the onset of introduction to solid food [60]. The cited studies indicated that although the production of leptin by neonatal rats was low, significant levels of leptin were detected in the stomach, which might suggest its exogenous source of leptin. Moreover, it was shown that leptin given orally to 4-day-old rats was absorbed intact by the immature gastric epithelium and transferred into the bloodstream. Leptin that was supplied to neonate rats inhibited food intake and downregulated endogenous production of leptin. The exact mechanism of leptin absorption has not been described; however, a feedback mechanism acting on gastric receptors has been postulated [61]. In addition to studies in animal models, it has also been shown that blood leptin levels are higher in breastfed infants than in formula-fed infants. Furthermore, the level of circulating leptin in breastfed infants positively correlated with the level of leptin in milk [37]. These results confirm that milk leptin can be absorbed and transported into the bloodstream and can exert a biological effect. During pregnancy, the placenta is the main source of leptin for the fetus, and after birth, breast milk represents a source of leptin for infants throughout the lactating period.

The conducted studies on the influence of leptin on the incidence of obesity in children do not give clear results. A number of these studies do not confirm the relationship between the content of leptin in breast milk and the prevalence of obesity in breastfed children. It seems that leptin may reveal many other beneficial effects on the developing organism.

## 7. Changes in the LEP Gene Sequence and the Risk of Obesity

In addition to environmental and maternal factors, genetic factors also play a role in the pathogenesis of overweight and obesity. Due to the multiplicity of these factors, it is extremely difficult to distinguish those that predispose infants and young children to obesity and overweight. A single factor increases the risk of a disease to a relatively small degree, but when numerous factors are involved, the risk of significant diseases increases [62].

It was shown that mutations and single-nucleotide polymorphisms (SNPs) in genes encoding adipokines and genes involved in energy balance may be associated with energy and appetite regulation imbalance. Much information on the importance of leptin in the pathogenesis of overweight and obesity comes from genetic studies. It is considered that sequence variants in the LEP gene may be involved in the pathomechanism of obesity development [63].

A recent study by Kroll et al. [64] showed the association of genetic variants in the LEP (rs7799039) and ADIPOQ (rs2241766) genes with body weight trajectories in children from birth to six years of age. They indicated that the ADIPOQ-rs2241766 TG and GG genotypes increased the risk of excess body weight in children from birth to 6 years of age and had a positive effect on body weight trajectories in girls. These findings were not confirmed in the boys’ group. Moreover, the LEP-rs7799039 genetic variant showed no association with the body weight trajectory in children [64]. Dasgupta et al. [65] also investigated the SNP variant rs7799039 and two others (rs2167270 and rs4731426) in the leptin gene. The results showed that these variants, both independently and in four haplotype combinations, were significantly associated with the risk of obesity in a South Indian population.

The genome-wide association study of BMI measurements at 12 time points from birth to 8 years of age (9286 children) in the Norwegian Mother, Father, and Child Cohort Study revealed that there are distinct molecular mechanisms that dynamically and specifically influence weight gain in infancy. The study identified a transient effect in the leptin receptor (LEPR) rs2767486 locus with no effect at birth and an increasing effect in infancy, peaking at 6–12 months of age, as well as a transient effect near the LEP gene (rs10487505) peaking at 1.5 years. Both signals were protein quantitative trait loci for LEP, which suggests key roles of variation in the leptin signaling pathway for healthy infant growth [66].

Other studies also indicated that the presence of sequence variants in the LEP gene is associated with higher levels of energy and total lipid intake, influencing the individual’s weight status [67]. The contribution of the genetic variant rs7799039 in the leptin gene as a marker of obesity is contradictory. On the one hand, a study in a pediatric population revealed no association between LEP-rs7799039 and obesity [68]. In adult populations, however, some studies revealed an association of this SNP with excess body weight [16,69,70].

Monogenic forms of childhood obesity are very rare. Leptin deficiency, however, can be a significant cause of this form of disease [71]. The study of El Saeed et al. [72] recently reported leptin gene variants in the coding region in 30% of a leptin-deficient group of 80 children who developed obesity during the first year of life [72]. In complex diseases such as obesity, the environment exerts an influence on the phenotype. The occurrence and frequency of SNPs which may predispose one to obesity may be associated with the given population in which research was conducted.

The duration of breastfeeding can reduce the risk of obesity in adulthood. Epigenetic regulation of the LEP gene may represent the mechanism underlying the protective effect of breastfeeding duration against obesity. The study of Obermann-Borst et al. [73] on a group of 98 children revealed that high BMI and leptin concentration were associated with lower methylation of LEP, while longer breastfeeding duration was associated with lower methylation of the leptin gene [73]. Another study on a group of 101 one-year-old infants demonstrated that higher LEP promoter gene methylation was observed when mothers breastfed their children for 7–9 months compared to those breastfeeding their babies for 1–3 months or for 10–12 months. The study also indicated that infant weight was significantly lower when children were breastfed for 10–12 months [74]. In the Isle of Wight Birth Cohort, a group of 259 infants at 10 years and one of 257 infants at 18 years were examined, and the breastfeeding duration was taken into account (exclusive breastfeeding vs. mixed feeding). The analysis of LEP gene methylation in these groups showed that total and exclusive breastfeeding duration was associated with DNA methylation of LEP. The duration of breastfeeding was also associated with the early transient overweight trajectory [75]. With the presented research results, it is worth noting that nutrition is one of the most important environmental factors that can influence early developmental processes, mainly through the regulation of epigenetic mechanisms during both pregnancy and neonatal periods. The maternal diet contributes to the establishment of the epigenetic profiles in the fetus, which can have a profound impact on susceptibility to certain diseases later in life, including the offspring’s obesity. Moreover, the maternal diet may also impact the neonatal microbiome, leading to specific epigenetic changes that may potentially predispose the child to the development of obesity at a later age [76,77,78].

Breastfeeding can help prevent overweight and obesity. However, many factors are involved in such complex diseases. The influence of the environment on changes in the human genome is still under investigation. The potential of LEP DNA methylation and the effect of breastfeeding on childhood obesity require further research in different populations and on larger sample sizes.

## 8. Conclusions

Due to a number of overlapping factors, the effect of breastfeeding on the health of a developing baby is multifaceted. It is difficult to estimate the effect of the individual components of breast milk. In the case of obesity, the occurrence of which is determined by numerous factors, estimating the influence of leptin, one of the breast milk components, is particularly challenging. Due to this, researchers around the world are obtaining different and often contradictory results. Therefore, we are not able to clearly define the effect of breast milk leptin on the prevention of obesity in breastfed children. The strength of the action of leptin also cannot be determined. Additionally, the concentration of leptin in breast milk changes during lactation and also differs on an individual basis. Nevertheless, the knowledge of how this hormone works and of its presence in breast milk suggests that it may have a beneficial effect on the health of babies, which is confirmed by some studies. Leptin’s influence on children’s body weight, growth rate or obesity later in life is not fully understood. The causes of obesity are very complex, and it is actually impossible to reliably determine the effect of a single factor, such as the presence of leptin in breast milk, on its prevention.

## Figures and Tables

**Table 1:** Variability of the concentration of leptin in human milk.

Source	Mean Leptin Value in Human Milk or Serum (Range)	Study Group (Abundance)	Method	Age of Fed Children
Bronský J. et al. (2006) [31] Chan D. et al. (2018) [32] Dares G. S. et al. (2019) [33]	0.50 (±1.37) ng/mL	56	ELISA (Biovendor-Laboratory Medicine)	1st week
	0.349 (0.031–3.968) ng/mL	430	ELISA (Meso Scale Discovery)	4th month
	0.640 (0.075–4.318) ng/mL 0.484 (0.055–6.576) ng/mL	135 125	ELISA (NO COMPANY)	1st month 3rd month
Fields D. A. et al. (2012) [3]	0.0918 (±0.0047) ng/mL	19	ELISA (NO COMPANY)	1st month
Karatas Z. et al. (2011) [34] Khodabakhshi A. et al. (2015) [23] Logan C. A. et al. (2019) [10] Miralles O. et al. (2006) [6]	BF * Foremilk: 0.33 (0.28–0.50) ng/mL Hindmilk: 0.40 (0.15–2.34) ng/mL	26	ELISA (DRG Instruments)	2nd month
	Milk of non-obese infant: 1.78 (1.67–1.94) ng/mL Milk of obese infant: 1.81 (1.65–1.94) ng/mL	40 40	ELISA (Mediagnost)	2nd–5th month 2nd–5th month
	388.8 (12–3941) ng/mL 269.6 (0.3–2077) ng/mL 320.4 (15.5–1475) ng/mL	668 445 69	ELISA (R&D Systems)	6th week 6th month 1st year
	Milk: 0.156 (±0.039) ng/mL Maternal plasma: 12.8 (±1.7) ng/mL	28	ELISA (R&D Systems)	1st month
Brunner S. et al. (2014) [35] Savino et al. (2002) [36]	Median (** IQR) Milk: 0.11 (0.19); maternal plasma: 9.52 (8.56) ng/mL Milk: 0.09 (0.18); maternal plasma: 8.30 (10.64) ng/mL	152 120	Milk RIA (Mediagnost); Maternal plasma ELISA (Mediagnost)	6th week 4th month
Savino et al. (2016) [37]	BF* Milk median (** IQR): 2.34 (5.73) ng/mL	23	RIA (Mediagnost)	<6th month
Schister et al. (2011) [38]	Median (range) 0.17 (0.08–0.25) ng/mL 0.11(0.06–0.20) ng/mL 0.15 (0.05–0.32) ng/mL	23 23 23	RIA (Mediagnost)	1st week 2nd month 6th month
Schueler et al. (2013) [39]	Foremilk: 0.9 (±0.7) ng/mL Hindmilk: 1.0 ± 0.8 ng/mL	12	RIA (Millipore)	2nd month
Uysal F. K. et al. (2002) [40]	Milk of obese infant: 0.27 (±0. 2) ng/mL Milk of non-obese infant: 0.37 (±0.4) ng/mL	17 33	RIA (Linco Research Inc)	2nd–4th month

* BF—breastfeeding group. ** IQR—interquartile range.

## Data Availability

The data presented in this study are available on request from the corresponding author.
